# Adaptation of Research Infrastructure to Meet the Priorities of Global Public Health

**DOI:** 10.3389/fbioe.2020.613253

**Published:** 2021-01-07

**Authors:** Rebecca L. Moritz, David R. Gillum

**Affiliations:** ^1^Colorado State University, Biosafety Office, Fort Collins, CO, United States; ^2^Environmental Health and Safety, Arizona State University, Tempe, AZ, United States

**Keywords:** research infrastructure, risk mitigation, high containment laboratories, SARS-CoV-2, research priorities, biosafety

## Introduction

The rise of SARS-CoV-2 has resulted in the urgent need to unlock the mechanisms behind the disease it causes, COVID-19, and to develop countermeasures. As a result, there has been a massive push to study the virus and to test potential candidate vaccines, therapeutics, and antivirals. This has caused a rush at many institutions to switch research priorities to contribute to knowledge about the virus and COVID-19. This means that biosafety professionals have been tasked with figuring out how to conduct research with a novel pathogen safely and securely, often without the requisite knowledge and experience to safely assess the risk. Due to the similarity to another coronavirus, SARS-CoV, many biosafety professionals use the risk mitigation measures for this virus as a starting point. Research with live SARS-CoV-2 requires biosafety level 3 (BSL-3) high containment with additional risk mitigation measures, such as specialized training, engineering controls, personal protective equipment, and health monitoring as well as being mindful of the communication complexities of an ever-connected world. Analyzing human samples, developing diagnostics tests, studying downstream metabolites or proteins, or conducting immunology assays may be performed in a BSL-2 facility with enhanced practices depending upon an institution's risk assessment and risk tolerance. Globally, there are regional differences in high containment laboratories and what is studied in each facility.

It is unknown if high-containment laboratory capacity in the United States meets or exceeds the national need or if they can be operated safely (Kingsbury, 2009). In addition, not all BSL-3 facilities are created equal and some do not have the optimal safeguards in place for conducting research with pathogens of pandemic potential. The Government Accountability Office (GAO) previously determined the absence of national standards for high containment laboratory design, construction, commissioning, operations, and maintenance raised concerns and increased the risk of laboratory accidents [US Government Accountability Office (GAO), 2013].

Some BSL-3 laboratories are built for specific purposes and others are built with a limited scope of use. Others are designed in an attempt to anticipate all future uses. More often than not, BSL-3 laboratories are value-engineered due to the high costs of construction, daily-operations, upkeep, and maintenance. Organizations do not want to overspend for features they might not use even though they might need them later on. As a result, there are a limited number of BSL-3 high containment facilities in the United States capable of conducting live SARS-COV-2 research safely and securely, especially with animal models.

In addition, not all researchers have the training and expertise to work inside of BSL-3 laboratories. Training programs vary greatly depending on the structure and expertise of the trainer and the previous experiences of researchers. Due to the different designs and resulting standard operating procedures (SOPs), often the training from one facility is not necessarily applicable to another. Although the principles and practices behind biosafety and biosecurity are the same regardless of the design, individuals must demonstrate competency in conducting experiments and the laboratory SOPs before working unescorted. In addition, the COVID-19 pandemic is an ever-evolving situation, and as a result, public health guidance is changing frequently. Currently, there is a greater risk of researchers being exposed to SARS-CoV-2 in the community than from working in the laboratory.

In combination, these factors present a challenging environment for biosafety professionals who facilitate research and ensure it is being performed in a safe and secure manner. It takes the entire institutional infrastructure—including but not limited to compliance and safety departments, facilities and maintenance personnel, oversight committees, information technology and security, and media relations—to ensure the research can be brought online swiftly and with limited hindrance in the most effective way possible.

## Perspective

### Risk Assessment

There is no such thing as no risk in research. The only way to ensure zero risk is to not conduct research. However, there are many ways to decrease risk and make it as close to zero as possible. The 5th Edition of Biosafety in Microbiological and Biomedical Laboratories (BMBL), published by the U.S. Department of Health and Human Services, describes the process for conducting a biological risk assessment in Section II and why it must be done by knowledgeable personnel.

Risk assessment is the process that enables the appropriate selection of microbiological practices, safety equipment, and facility safeguards that can prevent laboratory-associated infections (LAI) (US Department of Health Human Services, 2009; Gillum et al., 2016). This process is used to identify the hazardous characteristics of known and unknown infectious or potentially infectious agents or materials, the activities that can result in a potential exposure, the likelihood of exposure that will cause an LAI, and the probable consequences of infection (US Department of Health Human Services, 2009). All of this information is used to minimize the risks and protect the researchers, environment, and community. Ultimately, an institution must determine what its risk tolerance is and what risks are acceptable.

Many institutions have at least three different avenues for conducting risk assessments for research. The first is the knowledge and expertise of the researcher and laboratory personnel. The second is the formal review of the proposed research by a trained biosafety professional. The third is committee review by fellow researchers evaluating the research on behalf of the institution. Each assessment is unique and helps prevent blind spots in the process. However, it is not perfect. The risks can be overstated resulting in undue burden and/or expense or understated resulting in a variety of adverse consequences. The BMBL clearly states that if there is insufficient information to make a clear determination of risk, it is prudent to consider the need for additional safeguards until there is more data (US Department of Health Human Services, 2009).

While there can be a lack of information for novel pathogens, there is often an urgent need to study and understand them. Researchers, like many others, feel the need to contribute to society in a manner that is constructive and can help solve a challenge, such as SARS-CoV-2 and COVID-19. Yet, this could be a new area of research for them and they might not have the necessary knowledge and skill sets to work with the pathogen, whether they realize it or not. Also, many biosafety officers lack experience with viral research, let alone a pathogen like SARS-CoV-2, which requires working in high containment laboratories. Ultimately, it is the lead researcher's responsibility to ensure a safe working environment for their laboratory, and it may be necessary to seek expert knowledge outside the institution. In addition, the committee reviewing the research may be limited in scope because of its charge. Even if all three of these three review processes work well individually, there may still be communication gaps, resulting in a breakdown of the risk assessment process. This may increase the risk of an LAI or release to the environment or community.

### Risk Mitigation

There are many different institutions with wildly different organizational structures to manage research. From our experiences, each institution has their own way of doing things. This can complicate how risk mitigation is accomplished since there is no standardized structure.

Regardless of institutional structure, starting research with a novel pathogen is not something that should be taken lightly. Beyond the need to evaluate an investigator's scientific ability to conduct the research, there are other variables that need consideration and evaluation. The decision to conduct research with novel pathogens should be a partnership between the investigator and the institution, with close communication and collaboration with biosafety professionals. This will help ensure everyone is on the same page regarding safety and that all regulatory and compliance requirements are met.

The process and speed at which research can begin, is dependent on whether the investigator has proactive conversations with knowledgable parties (e.g., biosafety officer, occupational health, facilities personnel) to determine what is needed to conduct the research and obtain approvals. Risk mitigation has many different facets and may ensure the following:

- Laboratory is designed to contain the organism
- Facility has the proper engineering controls and monitoring
- Laboratory is equipped with the correct scientific equipment and devices
- Appropriate safety features are utilized
- Personnel have the requisite experience
- Personnel have safety and emergency response training
- Personal protective equipment and disinfectants are available
- Medical surveillance is followed
- Inactivation and decontamination procedures are utilized
- Institutional support obtained
- Community engagement in the research enterprise.

A major issue to consider is whether an institution already has the infrastructure necessary to safely conduct the research, or is it being started from scratch. Planning, designing, constructing, certifying, writing policies and procedures, and hiring personnel may take years. In addition, the risk mitigation process can take a significant amount of time to determine if the available infrastructure and personnel will truly mitigate the risk, especially in the case of novel pathogens.

### Public Health Consideration

Another piece of the risk mitigation puzzle—that for this situation deserves its own discussion—is public health considerations. For example, it is important to ask several questions when working with a novel pathogen, including:

- Are there any countermeasures effective against the pathogen?
- What arrangements does the institution have with public health agencies and medical providers?
- What procedures are in place if a researcher has an incident that could result in a LAI or develops symptoms of the pathogen?
- Is it possible to differentiate between viruses used in the laboratory vs. those circulating in the community?
- Should researchers be allowed to travel within the incubation period for the pathogen?
- When is a researcher at greater risk from the actions in their personal life than from work in the laboratory?

Relationships and collaboration with public health authorities, infectious disease physicians, occupational health personnel, and Clinical Laboratory Improvements Amendments (CLIA) certified diagnostic testing laboratories, are critical for the ability to test, quarantine, and treat researchers. The establishment of these collaborations takes time. Trying to address these issues during a pandemic is too late as these groups will be far too busy dealing with patients, logistics, and testing capacity. Building these relationships and developing trust is time consuming, requires buy-in from all groups, and needs to be tested with real-world scenarios.

### Ethical and Public Relations Considerations

Nowadays, people obtain their “news” from a wide variety of sources, some legitimate and some not. With the amount of misinformation and theories on social media regarding the origins of SARS-COV-2 and the COVID-19 pandemic, institutions must decide how much information to proactively share with the public. Consideration should be given to preparing a press release and talking points prior to beginning research, and assuredly before publishing the results. This will help ensure that the description of the research is accurate. It is also an opportunity to highlight the risk mitigation measures in place to ensure the public that the research is being conducted in a safe and secure manner.

In order to promote goodwill and trust with the community, it is important to be as transparent as possible when considering and conducting research with pathogens. It is also important to note that many institutions in the United States are subject to Federal Freedom of Information Act requests or other state open-records laws. Based upon the experiences of the authors, it is always best to be proactive when it comes to communication. Proactive and open communication allows the institution to better control the message, ensure everyone is on the same page, and use less resources to combat misinformation.

## Discussion

The appearance of SARS-CoV-2 and resulting COVID-19 pandemic has highlighted the difficulties with conducting research with novel pathogens in a crisis. There has been immense pressure to quickly obtain as much scientific information as possible. As a result, there has been a significant increase in the number of researchers who want to study SARS-CoV-2. Institutions must ensure that personnel doing the research are adequately vetted prior to beginning the work.

Research is not conducted in a vacuum and requires a significant amount of institutional support. However, research with pathogens of high consequence requires greater institutional resources. A proactive conversation with the right network of people at an institution will help to ensure there are not unnecessary roadblocks. The existence of an adaptable research infrastructure is critical when an institution needs to pivot to help address a pandemic. The training of researchers, experience of biosafety professionals and trainers, relationships with public health and medical providers, and foresight to anticipate potential interest from the media and general public is required.

Prior to the COVID-19 pandemic, there were institutions working safely with pathogens of pandemic potential. These laboratories were able to quickly transition efforts to gain a greater understanding of SARS-CoV-2 and develop countermeasures. While there are aspects of SARS-CoV-2 research that do not require high containment laboratories (e.g., rapid detection systems), ultimately a live virus model for SARS-CoV-2 is required to work in a high containment laboratory. Going forward, any future novel pandemic pathogens will likely require the use of high containment laboratories to study and develop countermeasures while mediating the risks to the research personnel, community, and the environment. The COVID-19 pandemic has shown us that it is important to react swiftly, in real-time, to better inform public health decisions. It is in everyone's best interest that institutions and researchers collaborate and work together to study emerging pathogens in an ethical and safe manner.

## Author Contributions

All authors contributed to the article and approved the submitted version.

## Conflict of Interest

The authors declare that the research was conducted in the absence of any commercial or financial relationships that could be construed as a potential conflict of interest.
